# Androgenesis-Based Doubled Haploidy: Past, Present, and Future Perspectives

**DOI:** 10.3389/fpls.2021.751230

**Published:** 2022-01-07

**Authors:** Brett Hale, Alison M. R. Ferrie, Sreekala Chellamma, J. Pon Samuel, Gregory C. Phillips

**Affiliations:** ^1^Molecular Biosciences Graduate Program, Arkansas State University, Jonesboro, AR, United States; ^2^Arkansas Biosciences Institute, Arkansas State University, Jonesboro, AR, United States; ^3^National Research Council Canada, Saskatoon, SK, Canada; ^4^Corteva Agriscience, Johnston, IA, United States; ^5^College of Agriculture, Arkansas State University, Jonesboro, AR, United States; ^6^Agricultural Experiment Station, University of Arkansas System Division of Agriculture, Jonesboro, AR, United States

**Keywords:** androgenesis, doubled haploidy, microspore culture, plant breeding, pollen

## Abstract

Androgenesis, which entails cell fate redirection within the microgametophyte, is employed widely for genetic gain in plant breeding programs. Moreover, androgenesis-responsive species provide tractable systems for studying cell cycle regulation, meiotic recombination, and apozygotic embryogenesis within plant cells. Past research on androgenesis has focused on protocol development with emphasis on temperature pretreatments of donor plants or floral buds, and tissue culture optimization because androgenesis has different nutritional requirements than somatic embryogenesis. Protocol development for new species and genotypes within responsive species continues to the present day, but slowly. There is more focus presently on understanding how protocols work in order to extend them to additional genotypes and species. Transcriptomic and epigenetic analyses of induced microspores have revealed some of the cellular and molecular responses required for or associated with androgenesis. For example, microRNAs appear to regulate early microspore responses to external stimuli; trichostatin-A, a histone deacetylase inhibitor, acts as an epigenetic additive; ά-phytosulfokine, a five amino acid sulfated peptide, promotes androgenesis in some species. Additionally, present work on gene transfer and genome editing in microspores suggest that future endeavors will likely incorporate greater precision with the genetic composition of microspores used in doubled haploid breeding, thus likely to realize a greater impact on crop improvement. In this review, we evaluate basic breeding applications of androgenesis, explore the utility of genomics and gene editing technologies for protocol development, and provide considerations to overcome genotype specificity and morphogenic recalcitrance in non-model plant systems.

## Introduction

In the vast majority of higher plants, a reproductive lineage is established by post-embryonic cell division that culminates with gametogenesis. Resultant gametes provide a basis for sporophyte-gametophyte life cycle alternation and allow the continuation of species through fertilization-mediated embryogenesis. In addition, plants have the capacity to undergo apozygotic embryogenesis (Soriano et al., 2013). This phenomenon is observed commonly during *in vivo*, asexual reproduction (e.g., recurrent and nonrecurrent apomixis; uniparental chromosome elimination) and is exploited *in vitro* (e.g., gametophyte culture; somatic embryogenesis) for the genetic improvement of commercially-relevant species (Ferrie and Caswell, 2011). The most efficient method for artificially-induced, gametophyte-based apozygotic embryogenesis is androgenesis – a developmental program characterized by the generation of male-derived haploid progeny.

**Figure 1:**
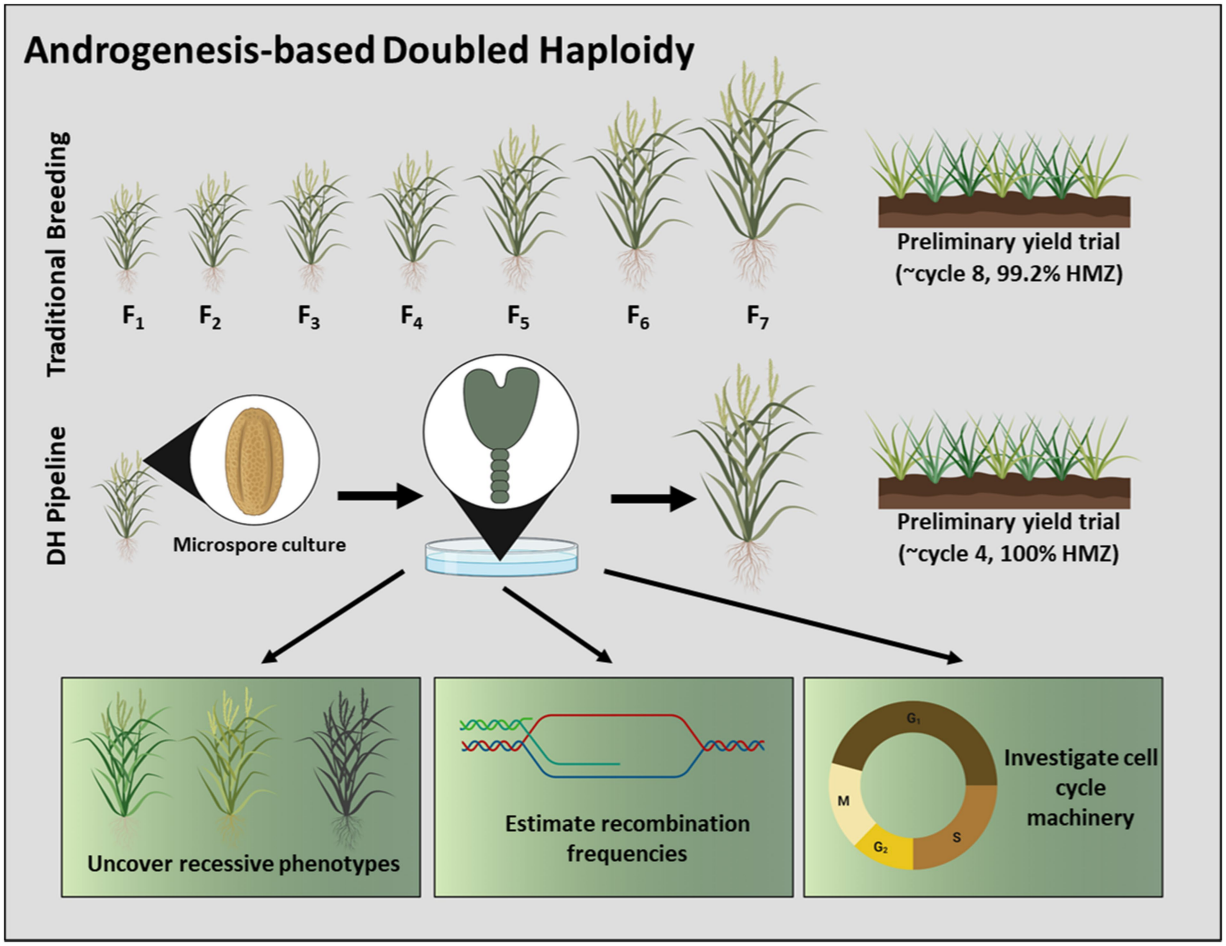
Schematic overview of androgenesis-based doubled haploidy and its uses in plant breeding. A DH pipeline has the capacity to advance cultivar development in a timeframe unmatched by traditional methods. The nature of microspore-derived DH’s permits their exploitation to uncover recessive phenotypes, to estimate recombination frequencies, and to investigate cell cycle machinery, among other benefits. Note that wheat (*Triticum aestivum*) is depictive of all species, and the number of cycles is plant-specific. Moreover, this illustration considers *in vitro* embryogenesis to be a cycle in the breeding process.

The primary benefit of androgenesis is the recovery of genetically-fixed, doubled haploid (DH) tissues, derived by leveraging the haploid state of the microspore and subsequent genome doubling. Given their homozygous nature, DH plants are ideal for rapid cultivar development in self-pollinated crops (Figure 1), for the generation of inbred parental lines during F_1_ hybrid production, and for the discovery and introgression of novel traits (Ferrie and Möllers, 2011; Germanà, 2011). In the same context, DH technology has the potential to reduce undesirable events that may arise during the breeding pipeline, such as self-incompatibility during hybrid development and hemizygosity during transgenesis and genome editing (Dwivedi et al., 2015; Niazian and Shariatpanahi, 2020). Androgenesis-based doubled haploidy also serves as a model for fundamental research. The microspore is derived post-meiosis; thus, microspore-derived androgenic plants are useful for the construction of linkage maps, estimation of recombination frequencies, and for the manipulation of cell cycle machinery (Figure 1; Touraev et al., 1997; Ferrie and Möllers, 2011). Androgenesis also provides a tractable approach for the study of cell fate determination that may be extended to somatic and zygotic embryogenesis platforms (Hale et al., 2021).

Techniques for *in vivo* androgenic induction have been discussed recently (Ren et al., 2017); thus, this review focuses primarily on *in vitro* haploidization, its application in commercial plant breeding systems, and potential methods to expand protocols to recalcitrant species. In doing so, we emphasize the value of single-cell based approaches for improved selection efficiency and cultivar development, highlight advances in gene product delivery systems to drive cell fate redirection, and consider ‘omic’ and tissue culture-related strategies for improved androgenic response.

## Plant Breeding Applications: From Lab to Field

In a conventional, science-based plant breeding process, genetic variability is created by cross-pollinating different parents with desirable trait combinations, followed by continuous selection and selfing. In traditional plant breeding strategies, such variability was evaluated, and selections based purely on the observed phenotype of the individual entries (Breseghello and Coelho, 2013). Later, with the development of molecular breeding techniques, selection of plants was fast-tracked using molecular markers linked to the desirable traits. Genetic variation was also generated using techniques such as mutagenesis, so that desirable traits were incorporated and later, selection was done to identify and select those recombinants (McNally et al., 2009). Since reducing timelines was a major goal in improving the plant breeding process, speed breeding was later introduced as a technique to reduce the total time to develop improved plant varieties (Ahmar et al., 2020). In this context, DHs allow homozygous inbred lines to be developed much faster and more efficiently than traditional breeding methods (Jarrod, 2020). Combining DHs and marker-assisted selection (MAS) has resulted in a significant advancement of the breeding systems in many major crops (Tuvesson et al., 2007). Genomic selection (GS), as a recently emerged technology to predict the performance of plants without phenotyping, has proven to be effective in plant breeding (Li et al., 2020; Krishnappa et al., 2021).

DH methods have been, and are being, used to accelerate the breeding programs in a variety of crop plants, including maize (*Zea mays*), barley (*Hordeum vulgare*), *Brassica* sp., wheat (*Triticum aestivum*), and several vegetable crops (Ho and Jones, 1980; Ferrie and Möllers, 2011; Krishnappa et al., 2021). Maize is a crop where such an increase in improvement efficiency has resulted in a significant increase in genetic gain (Li et al., 2020). Geiger et al. (2013) reported that genetic variances and *per se* performance were substantially increased in haploids and DH in maize. This might have significantly contributed to progress in maize hybrid and population breeding. Wheat is another species where the use of DH technology has significantly reduced the breeding timelines and increased genetic gain (Barkley and Chumley, 2012). *Brassica* sp. was identified as one of the best responding species for androgenesis. Most of the currently grown *B. napus* varieties originated from DH technology and many of the *B. oleracea* vegetable breeding programs also use doubled haploidy (Ferrie and Möllers, 2011). In vegetable crops such as cauliflower (*Brassica oleracea* var. *botrytis*), a high degree of cross-pollination and strong S-allele results in enormous heterozygosity. This makes conventional selfing for the development of homozygous inbreds practically impossible (Nieuwhof, 1963). Therefore, the development of complete homozygous lines through the androgenesis process remains very attractive. In crops like cassava (*Manihot esculenta*), genetic improvement is slow due to long breeding cycles, large genetic load, and the heterozygous nature of parents and progeny (Ceballos et al., 2015). In such circumstances, the development of a DH system would greatly benefit breeding efforts.

The use of DHs in the breeding process was originally determined based on the plant mode of reproduction. In crops that are self-pollinated, DHs can be used directly as the final varieties/cultivars. Homozygosity created by the DHs allows the selection of both qualitative and quantitative characters (Murovec and Bohanec, 2012). Self-pollination allows further propagation of these cultivars as true-breeding lines. DHs can also be used as parental lines or test-cross lines in the hybrid breeding of cross-pollinated species (Rudolf-Pilih et al., 2019). Additionally, they can be used in a recurrent selection scheme in which superior doubled haploids of one cycle can be used as parents for the next hybridization cycle (Murovec and Bohanec, 2012). More recently, DHs have been recommended as breeding material in reverse breeding-based combining ability tests (Rudolf-Pilih et al., 2019) to enhance the hybrid breeding process.

Hybrid breeding systems have replaced conventional variety development in many major crops of economic value, irrespective of their mode of reproduction. This is due primarily to rapid advancement in the development of hybridization systems (Nienhuis and Sills, 1992; Murovec and Bohanec, 2012; Mette et al., 2015; Hooghvorst et al., 2020). There are inherent advantages of using DHs in a hybrid breeding program, e.g., the creation of genetically distant, distinct, pure, inbred lines and their combination for the highest possible combining ability (Murovec and Bohanec, 2012; Dezfouli et al., 2019). Thus, the advancement of DH platforms has become a focal point in hybrid breeding systems for many crops of interest (Begheyn et al., 2016). DHs have more relevance in perennial crops or outcrossing plants with severe inbreeding depression (Seeja and Sreekumar, 2020).

There are two different approaches for creating DH lines. These include androgenic and gynogenic methods (Forster et al., 2007). From a breeding perspective, both systems have their own advantages and disadvantages. When obtained in a genotype-independent manner with high efficiency, the androgenic system provides some distinct advantages. For example, androgenesis provides an agile platform for the incorporation/complementation of emerging genomic technologies (explained in different sections within this article). While androgenesis and gynogenesis methods are employed commercially for genetic gain, this review emphasizes the use of androgenesis-based doubled haploidy in a plant breeding program.

### Major Applications of DH in Plant Breeding

In short, doubled haploids have enormous potential to improve agronomic characters in crop plants (Krishnappa et al., 2021). Breeding/selection approaches that allow a rapid increase in selection intensity, genetic diversity in the breeding population and/or heritability of traits, and a reduction in the length of the breeding cycle are needed to obtain higher genetic gain in breeding programs (Cooper et al., 2014). Described below are some ways that DHs drive such genetic gain. It is noteworthy that, in addition to the outlined methodologies, haploids and doubled haploids contribute to several other components in a seed product development pipeline.

#### Create Variability/A Greater Number of Recombinants That Can Be Selected

DHs provide a unique opportunity to create a large number of fixed recombinants simultaneously, allowing selection within the same time window and environment. Compared to gynogenic methods, where the total number of recombinants from a single event is limited, the androgenesis system (microspores) provides the flexibility to generate a large number of recombinants. Tadesse et al. (2012) reported that, theoretically, if a hybrid has *n* pairs of independently segregating genes, the chance to select a particular homozygous genotype from the F2 population in a conventional breeding program is (1/2)2*n*, whereas in haploid breeding, it is (1/2)*n* (Chu, 1982). In other words, the selection efficiency in haploid breeding is 2*n* times greater than that of conventional methods.

Bradshaw et al. (1995, 1998) reported that a larger population allowed the detection of quantitative trait loci (QTLs) of smaller effect. In general, to improve quantitatively-inherited traits such as yield, it is highly important to increase the population size by nominating a limited number of elite x elite crosses for DH production (Tadesse et al., 2012). It is also important to note that linkage between genes plays a role in determining the genetic recombination for selectable traits (Choo, 1981). Therefore, as more traits are considered, a higher number of starting recombinants results in a more desirable combination of traits.

#### Reduce Time, Space and Efficiency for Breeding

Urgent development of new cultivars is required to meet the demands of an increasing population and challenges of a changing climate, yet cultivar development is a lengthy and time-consuming process (Dwivedi et al., 2015). Comparing conventional and DH wheat variety development, Barkley and Chumley (2012) predicted that a 150% increase in yield potential may be achieved with the use of DH. In addition, they also estimated a 4-year reduction in variety development using the DH method for the same overall outcome at the end of the breeding cycle. Similarly, for sorghum (*Sorghum bicolor* L.), a DH breeding system allows parental lines to be developed in 2 years, where it takes up to 7 years to complete similar developments conventionally (Hussain and Franks, 2019).

Availability of novel technologies has further enabled reduction in the breeding time, or an improved genetic gain using DHs in a breeding program. For example, through the development of DH wheat lines containing rust resistance genes, Wessels and Botes (2014) showed that integration of MAS and DH technology into conventional breeding processes could increase the speed of cultivar development. Genome sequences of many food crops are available today and can be readily leveraged in any breeding program, in addition to the information on markers and genomic regions associated with agronomically beneficial traits (Dwivedi et al., 2007, 2015; Varshney et al., 2009, 2013; Thudi et al., 2012). Development of abundant genomic resources (Dwivedi et al., 2007), high throughput, cost-effective phenotyping (Cobb et al., 2013; Fiorani and Schurr, 2013; Araus and Cairns, 2014), and genotyping tools (Varshney et al., 2009; Thudi et al., 2012, 2020) contributes to the enhancement of breeding selections from DHs. This allows the best opportunity to use scientific knowledge generated through DH technology for several years in crops [e.g., barley, *Brassica* sp., maize, rice (*Oryza sativa*), and wheat]s, and integrate them with phenomics and genomics to accelerate breeding pipelines. Li et al. (2020) simulated DH-based genomic selection procedures and reported that it is possible to obtain substantial long-term genetic gain along with the feasibility of increasing multiple traits simultaneously (Brauner et al., 2019).

If *n* loci are segregating, the probability of getting the desirable genotype is (1/2)*^n^* by the haploid method and (1/4)*^n^* by the diploid method, due to the occurrence of additional segregating progenies compared to the distinct allelic families (Badu et al., 2017). Therefore, as the number of traits that are considered in a breeding process increases, the efficiency of the doubled haploid method increases. Mayor and Bernardo (2009) studied GS and MAS in DH versus F2 populations and found that GS was superior to MAS and DH populations were superior to F2-derived populations using GS (Ren et al., 2017).

Large numbers of finished inbred lines are attained at once in a DH-based breeding pipeline compared to multiple years and stages in a conventional method. This eliminates the need for handling larger numbers of breeding materials from different generations of inbreeding. In other words, significant field resources are saved by allowing smaller population sizes to produce a homozygous (fixed) trait, and/or evaluation of better performing lines (Park et al., 1976; Ho and Jones, 1980; Dwivedi et al., 2015; Li et al., 2020). In addition to the advantage of evaluating all the possible recombinants together, logistics like shipping the seed, managing inventories, planting nurseries, selfing, and maintaining lines are simplified (Röber et al., 2005; Prasanna et al., 2012; Chaikam et al., 2019). Simplified logistics in overall operations lead to significant cost savings in the long term (Jumbo, 2010).

#### DHs Provide Excellent Recombinant Inbred Lines for Molecular Mapping Applications and Trait Stacking

DHs can be repeatedly tested to recover reliable data in multiple generations, owing to their uniform genetic identity, and provide high value in quantitative and qualitative trait mapping (Yang et al., 2020). Complete homozygosity of DH lines offers a higher phenotype to genotype correlation, thereby facilitating better estimation of QTL effects in marker-trait association studies (Hyne et al., 1995). In addition, DH lines are expected to improve the selection response compared to F2 populations when dealing with complex traits of low heritability controlled by many QTLs (Mayor and Bernardo, 2009) based on simulation studies in marker-assisted recurrent selection and genome-wide selection. The ability to generate DHs enabled the establishment of chromosome maps in a range of species, such as barley, rice, rapeseed, and wheat (Forster and Thomas, 2005). In addition, it also provided most mapped genetic markers, >90% in some species (Forster et al., 2007). Due to low population structure and quick decay of linkage disequilibrium DH lines derived from landraces were proposed to be ideal for association mapping (Strigens et al., 2013; Melchinger et al., 2018). DHs are also of high utility in establishing marker–trait associations in bulked segregant analysis (BSA; Michelmore et al., 1991). Several disease and pest resistance breeding traits, as well as quality traits, were established using marker-assisted selection in BSA using DHs (Forster et al., 2007; Melchinger et al., 2018). Therefore, integration of DHs with MAS provides an excellent opportunity to maximize selection gains and accelerate development of crop cultivars (Belicuas et al., 2007). In genomic selections, the genome-wide marker data along with phenotyping are used to estimate genomic estimated breeding values (GEBVs) for predicting performance. In comparison to MAS, genomic selection incorporates all marker information, thereby avoiding biased marker effect estimates and capturing more of the variation due to small-effect QTL (Dwivedi et al., 2015). This information is then used to predict performance of a data set where only genomic information is available (Heffner et al., 2009). Predictions for GEBV can be done without phenotypic characterization, which enables breeders to make early selection decisions and may shorten breeding cycles (Daetwyler et al., 2015). Purely homozygous nature of DHs provides an excellent opportunity to increase accuracy in genomic predictions.

Expressed sequence tags (ESTs) can be used as a tool to identify genes controlling a certain trait and map them to a chromosomal location associated with said trait (Wang et al., 2001). In addition, co-locating markers/traits and their associations can be verified (Badu et al., 2017). DHs play a vital role in integrating the genetic and physical maps. This enables precision in targeting candidate genes (Künzel et al., 2000; Forster et al., 2007). Another way to link genes and phenotypes is to induce mutations and analyze their phenotypes (Szarejko and Forster, 2007) for forward and reverse genetics approaches, where use of a DH line will help in increasing precision (Forster et al., 2007; Badu et al., 2017). Doubled haploids are also useful in rapid isolation and purification of selected mutants in subsequent generations.

#### Assessing Effect of Environment on the Expression of Traits

DHs provide the best material for assessing gene expression across different environments when compared to early selfing generations (Foiada et al., 2015; Yan et al., 2017). Strigens et al. (2013) showed that DH lines from landraces and open-pollinated varieties (OPVs) could be evaluated in replicated trials with high precision, which is not possible when using landraces and OPVs due to their high heterogeneity. Moreover, a high genetic load of deleterious alleles and high heterogeneity prevented the use of landraces and OPVs in hybrid maize breeding (Wilde et al., 2010; Melchinger et al., 2018). Due to the occurrence of a single chromosome during haploid generation, deleterious alleles are readily expressed and eliminated through natural or artificial selection.

#### Ease With Variety Registration/Protection

The plant variety protection act (USDA) provides intellectual property protection for new varieties of plants, giving owners 20-plus years of exclusive rights. An application for Plant Variety Protection requires submission of seed/plant material, which will be assessed for novelty, distinctness, uniformity, and stability. It is unlikely to develop conventional inbred lines with 100% homozygosity or homogeneity. Consequently, residual heterozygosity in these conventional inbred lines can sometimes delay plant variety registration (Chaikam et al., 2019). The aforementioned criteria of a variety can be easily established due to the complete homozygosity of DH lines, making variety registration/protection relatively easy (Röber et al., 2005), which in turn results in reduced time to commercialization (Bordes et al., 2006; Chaikam et al., 2019).

#### Use in Genetic Transformation

Use of haploid tissue in genetic transformation has been previously discussed (Swanson and Erickson, 1989; Chauhan and Khurana, 2011; Brew-Appiah et al., 2013; Shen et al., 2015). Integration of the transgene at a haploid state would allow creation of homozygous transgenic events with 100% pure genetic background. This facilitates stable fixation of the integrated gene (Chauhan and Khurana, 2011). Transformation can be done using any type of haploid plant tissue (Murovec and Bohanec, 2012), but microspores provide an ideal single cell and have proven to be feasible in many species (Eudes and Chugh, 2008; Chugh et al., 2009). For species where germplasm response to androgenesis and transformation are low, trait introgression will be significantly benefited by haploid transformation. For single gene introgression, the expected probability of individuals with a desired homozygous genotype is 1/4. If *n* represents the number of independently segregating genes, the frequency of expected genotypes decreases exponentially following the formula 1/4*n* (Lübberstedt and Frei, 2012; Shen et al., 2015). Haploid target genotypes are generated with a frequency of 1/2*n*, and thus significantly reduces population size required to achieve successful genetic combinations (Ren et al., 2017).

#### Androgenesis From Single Cells Can Be Used as a Source of Artificial Variability Creation

Induced mutations have been used mainly to improve particular characters in well-adapted local varieties or to generate variation, which is difficult to be found in germplasm collections (Ferrie and Möllers, 2011; Germanà, 2011; Geiger et al., 2013). About 70% of developed mutant cultivars were released as direct mutants, i.e., without any further breeding; the remaining 30% were developed through cross-breeding programs (Maluszynski et al., 2000). DH systems have many attractive benefits for inducing, selecting, and fixing mutations (Szarejko and Forster, 2007). Haploid cells provide an ideal target for mutation induction and selection, for the screening of both recessive and dominant mutants in the first generation after mutagenic treatment, for the avoidance of chimerism, and for the shortening of breeding times. Induced mutations are predominantly recessive and are normally selected in the second or third (M2 and M3) generation after mutagenic treatment. Mutation of haploid cells enables the immediate expression of recessive mutations and recovery of homozygous diploids by chromosome doubling (Howland and Hart, 1977). Application of DH systems enhances the effectiveness of the selection of desired recombinants, shortening the time required for mutation detection and evaluation (Forster and Thomas, 2005; DePauw et al., 2011). If the mutagenic treatment is carried out on quiescent seed and the M1 plants are used as gamete donors for DH production, which saves many generations normally needed to produce pure breeding lines. Another option is *in vitro* mutagenic treatment of haploid cells. If microspores are used in this option, it works best if the mutagenic agent is applied at the uninucleate stage, before the first nuclear division, in order to avoid heterozygosity and/or chimerism caused by spontaneous diploidization through nuclear fusion. *In vitro* mutagenic treatment can also be accompanied by *in vitro* selection of desired traits.

#### Ploidy-Based Breeding Strategies

Polyploid organisms may often exhibit increased vigor and, in some cases, outperform their diploid relatives in several aspects. In many species, improved plant cultivars were developed for the increment in plant organs (“gigas” effect), buffering of deleterious mutations, increased heterozygosity, heterosis (hybrid vigor), high yield, improved product quality, tolerance to both biotic and abiotic stresses, and as a tool for gene transfer in interspecific crosses (Sattler et al., 2016). DHs can be used as a tool in enabling ploidy breeding strategy in crops where a DH system is possible (Bhatia et al., 2017). In a cauliflower breeding program, it was reported that tetraploid lines had more than a 50% economic yield increased as compared to the diploids and exhibited normal fertility. A triploid hybrid was produced by using these tetraploid lines as the pollen parent with a diploid cytoplasmic male sterility (CMS) line as the female (Bhatia et al., 2017). In cauliflower, heterosis is very low because of a narrow gene pool (Nieuwhof, 1963). Therefore, ploidy breeding may serve as a better strategy to the conventional breeding program. Chromosomal stability of the tetraploid lines (Szadkowski et al., 2011) needs to be worked out before their long-term use.

In fruit crops, DHs offers specific opportunities to manipulate seed size with ploidy (Germanà, 2011). Another potential avenue is to create seedless triploids (Germanà, 2009). In papaya (*Carica papaya* L.), gametic embryogenesis, particularly obtained *via* anther culture, seemed to contribute directly to the production of female pure lines (Rimberia et al., 2006).

#### Landraces and Diversity Inclusion

DH technologies open new opportunities for characterizing and utilizing the genetic diversity present in GenBank accessions. This is well-illustrated in maize, where heterogeneous populations of landraces helped in unlocking genetic diversity (Wilde et al., 2010). Their genetic heterogeneity and heavy genetic load are two distinct reasons for the limited use of landraces in breeding programs. These limitations may be overcome by *in vivo* DH techniques (Strigens et al., 2013). Wilde et al. (2010) reported that large genotypic variances among DH lines derived from landraces allowed the identification of DH lines with grain yields comparable to those of elite flint (EF) lines, enabling selected DH lines to be introgressed into elite germplasm without impairing yield potential. Large genetic distances of DH lines derived from landraces help to broaden the genetic base of the EF germplasm. Due to the low population structure and rapid decrease of linkage disequilibrium within populations of DH lines landraces, these would be an ideal tool for association mapping. However, Zeitler et al. (2020) reported that landrace DH lines may result in a decreased genetic diversity.

## Applications for Gametophyte Reprogramming

As described in previous sections, androgenesis is a biological process that culminates in an entity linked entirely to an individual male gametophyte. Conventionally, this inferred spontaneous, *in vivo* haploidy in which a fertilized embryo sac contained an inactivated female nucleus, resulting in an embryo with an exclusively paternal genetic program (Kermicle, 1974). More accurately today, the definition of androgenesis is expanded to *in vitro* male-derived haploidy that can be induced in the laboratory through multiple approaches, including (i) anther culture; (ii) isolated microspore culture; (iii) shed microspore culture; and (iv) meiocyte-derived callogenesis (Seguí-Simarro, 2010). These androgenic pathways differ oftentimes in the induction stage but lead to the same final haploid or doubled haploid product. Microspore embryogenesis is widely studied and applied (discussed elsewhere in this chapter). The developmental ontogeny of microspore embryogenesis typically follows a division pattern similar to zygotic embryogenesis (Zaki and Dickinson, 1991; Maraschin et al., 2005b; Supena et al., 2008); however, in some species, the sequence of differentiation involves several randomly oriented initial cell divisions that resemble sporophytic growth (Radoeva and Weijers, 2014). Subsequently, a well-defined protoderm can be recognized, and is the marker for embryo formation. At this stage of development, compact masses with a protoderm are defined as embryo-like structures (ELS) that give rise to an embryo with all primary tissues found in zygote-derived embryos (Soriano et al., 2013). It is unclear whether the competence to undergo microspore embryogenesis generates a reproductive edge or whether parallel processes occur naturally. Alternatively, given the derivation of embryogenesis from spore-like evolutionary precursors (Taylor et al., 2009), microspore embryogenesis may be a remnant of an essential developmental potential. Although the molecular mechanisms for induction have yet to be elucidated, it was recently shown that chromatin regulation using Trichostatin-like compounds contributed to developmental control (Li et al., 2014). However, the universal application of epigenetic additives (and other exogenous biomolecules) across crop species and genotypes to trigger androgenesis-based embryogeny remains to be realized, and attempts of expanding methods across genotypes and taxa are in progress (see succeeding text). This section focuses on the use of molecular manipulation *in vitro*, resulting in the induction of androgenic events and targeted mutagenesis using DNA-free transduction approaches.

Embryogenesis-relevant genes can be used ingeniously to trigger microspore de-and redifferentiation and are expected to provide much-needed DH methods. DNA-free delivery of gene products could enable the adoption and expansion of DH platforms across species and genotypes (Sinha et al., 2021). Moreover, regulating androgenesis-associated gene expression by internalizing exogenously supplied molecules [e.g., RKD Transcription activator-like effector (TALE) proteins in wheat] has potential to enhance androgenic response and ultimately accelerate plant breeding programs (Barkley and Chumley, 2012). Sinha et al. (2021) analyzed RKD promoters from wheat and triticale (× *Triticosecale*) and custom-synthesized a TaRKD-TALE protein for delivery using a cell penetrating peptide (CPP) Nano-carrier. The purified protein was covalently conjugated with R9 CPP, (Cys (Npys)-(D-Arg)9), and successfully transduced into wheat microspores. The embryogenesis-related marker genes early-culture abundant (ECA1), RWP-RK domain-containing proteins (RKD1), and Tapetum determinant 1 (TPD1) were upregulated significantly in the TALE-transduced microspores compared to the control. In addition, microspores cultured after the transduction of the R9-TaRKD-TALE protein yielded a considerably higher number of ELS and green plants in wheat cultivar, AC Fielder. The researchers also reported that microspore quality was not affected by the internalization of R9-TaRKD-TALE; on the contrary, transduced microspores showed a higher recovery post 120 h of culture. This work demonstrated the successful deployment of the TALE protein to improve the androgenesis process, and has potential for expansion to other crops/genotypes in the future.

Recently, Bélanger et al. (2020) investigated the role of miRNA for developmental plasticity and cell fate reprogramming within the barley microspore. Their findings indicated that the switch toward embryo differentiation involved miRNA-directed regulation of members of the ARF, SPL, GRF, and HD-ZIPIII transcription factor families. Approximately 41.5% of these targets were shared between day-2 and day-5 microspores, while 26.8% were unique to day-5 microspores. The former set may disrupt transcripts driving the destined pollen development, while the latter group may direct the microspore toward embryogenic pathways. There has been diligent research investigation into the epigenetic regulation of androgenesis, including the exploration of DNA methylation (El-Tantawy et al., 2014), histone methylation (Berenguer et al., 2017), acetylation (Li et al., 2014), and the upregulation of embryogenesis genes by TaRKD-TALE (Sinha et al., 2021). While these mechanisms are capable of disrupting gametophytic gene expression, they are not capable of degrading transcripts altogether. This further suggests a role for small regulatory RNAs (sRNAs) in microspore dedifferentiation. In wheat, increased expression of 24-nucleotide (nt) sRNAs was correlated with the progression of an embryogenic program, while sRNAs ≥23-nt demonstrated an opposing expression pattern (Seifert et al., 2016). Furthermore, sRNAs play essential functions in key developmental processes, including embryo, leaf, and meristem patterning and flower development (D’Ario et al., 2017). Thus, it is possible that *in vitro* modulation of sRNA expression may help drive the gametophyte to sporophyte transition.

Microspore embryogenesis is employed for practical breeding purposes and genetic mapping in many species; yet, is restricted oftentimes to a few responsive genotypes. A deeper understanding of the molecular mechanisms controlling gametophyte totipotency and subsequent histodifferentiation are needed to overcome species and genotypes dependencies. A combination of genomic (e.g., next-generation sequencing and network/meta-analyses) and biotechnological approaches could help establish more universal androgenesis induction protocols in the foreseeable future. Additional applications for gametophyte reprogramming are described by Dwivedi et al. (2015).

## Expansion of Protocols Across Genotypes and Taxa

Development of genotype-independent DH methods and protocols are essential for breeding purposes. However, this is not always possible during androgenesis, as genotypic differences do occur and in some cases differences between plants within the same genotype also exist. Several DH methods have shown that genotype has no or a limited role in DH response, for example, haploid inducer lines (Jacquier et al., 2020) or CENH3 (centromere variant of histone 3). These methods are currently not available for all species and are not discussed in this review.

In many species, embryogenic lines have been identified and these are used as model genotypes to conduct genetic, genomic, biochemical, or physiological experiments. Microspore embryogenesis is generally regarded as routine in *B. napus* but differences in embryogenic response do exist among genotypes. The highly embryogenic line Topas DH4079 (Pechan and Keller, 1988; Ferrie, 2003), which has been used as the model in countless experiments, is derived from the cultivar Topas, which is a poor responding cultivar in terms of microspore embryogenesis. Embryogenic lines are also available in other *Brassica* species (Ferrie et al., 1995; Hiramatsu et al., 1995; Barro and Martín, 1999; Lionneton et al., 2001).

### Embryogenic Biomarkers

Over the years, many DH protocols have been developed by systematically evaluating the different factors that influence microspore embryogenesis (donor plant conditions, genotype, developmental stage of the microspore, pretreatments, media components, and culture conditions). More recent studies are utilizing ‘omics’ methods and targeting the genes and the biochemical processes that trigger the switch from microspore development to embryo development.

Over the years, simple markers like morphological or physiological characteristics have been identified to denote induction or early embryogenesis. An observed indicator is the “star-like” morphology (Indrianto et al., 2001; Maraschin et al., 2005a) which occurs when the nucleus moves to the center of the cell and the vacuole splits into fragments.

It has also been shown that the identification of certain genes can indicate an embryogenic response. In *Brassica*, genes such as BABY BOOM (Boutilier et al., 2002), LEC2 (Malik et al., 2007), SERK1, and SERK2 (Ahmadi et al., 2016) have been shown to be involved in the steps of microspore embryogenesis and subsequent plant regeneration. The expression of LEC2 was useful in distinguishing embryogenic and non-embryogenic cultures after 3 days (Malik et al., 2007). SERK1 was up-regulated during the first few days of culture (day 1–5) and then decreased from globular to torpedo embryo stages. However, SERK2 expression levels increased throughout the early stages of embryogenesis and through to regeneration (Ahmadi et al., 2016). BABY BOOM and AINTEGUMENTA-like 5 (AIL5) were also identified in wheat when comparing freshly isolated microspores, microspores exposed to a pretreatment, and after 8 days of culture (Seifert et al., 2016).

Omics methods (e.g., transcriptomics, proteomics) can open up new avenues to understand the mechanism of microspore embryogenesis. In these experiments, there is a requirement for comparing a cultivar or culture treatment that is responsive with a cultivar or condition that is not. This is not always possible in the recalcitrant species, hence most of the work has been done in *Brassica*, barley, or wheat. There are only a few studies that have looked at protein changes during the different steps of the *Brassica* DH process. Early studies identified heat shock proteins (HSPs) associated with cell proliferation (Pechan, 1991). HSP synthesis has been reported during stress-induced microspore embryogenesis across many platforms (Cordewener et al., 1995; Telmer et al., 1995; Zarsky et al., 1995; Bárány et al., 2001; Seguí-Simarro et al., 2003); however, it remains unclear if HSP activity coincides primarily with the onset of embryogenesis or cytoprotection (Zhao et al., 2003; Testillano, 2019; Hale et al., 2020). In another study, cabbage (*B. oleracea* L. var. *capitata* L.) microspores were subjected to the high temperature pretreatment of 32°C as well as the 25°C untreated control (Su et al., 2020). The authors were able to identify 97 differentially expressed proteins found in the highly responsive embryogenic line at 32°C but not in the recalcitrant line. These proteins could then be classified based on their function, that being carbohydrate metabolism, protein synthesis and degradation in the endoplasmic reticulum, signal transduction, and cutin, suberin, and wax biosynthesis (Su et al., 2020).

The pollen transcriptome has been described for several species (soybean, rice, maize) and has been the subject of several reviews (Rutley and Twell, 2015; Liu and Wang, 2021). However, there are limited publications on transcriptomics in microspore embryogenesis. In wheat, the different stages of microspore embryogenesis have been analyzed using transcriptome and small RNA sequencing (Seifert et al., 2016). The authors were able to identify up-regulated genes that were involved in DNA methylation, histone methylation, and histone deacetylation. In barley, 96 differentially expressed EST’s were identified when freshly isolated microspores were compared to pretreated microspores (4°C). These encoded genes were involved in protein degradation, starch and sugar hydrolysis, stress response, and cell signaling (Maraschin et al., 2006). In a more recent study in barley (Bélanger et al., 2018), microspores at day 0, 2, 5 were compared. It was determined that there were approximately 14 K genes, with 3,382 differentially expressed genes between the microspore populations. This corresponded to the up-regulation of genes associated with glutathione s-transferase and heat shock proteins and the down-regulation of ribosomal subunit protein genes. From day 2 to 5, there was an induction of genes involved in early embryogenesis, hormone biosynthesis, hormone signaling, and secondary metabolism. Gene expression profiling in temperature-stressed soybean microspores reinforced these findings, suggesting that cellular reprogramming of the microspore entailed a traditional stress response, suppression of gametogenesis-relevant transcripts, and an increase in genes involved in cell division and proliferation (Hale et al., 2020).

As demonstrated, “omic” methods are beneficial in trying to tease out the components or genes involved in microspore embryogenesis; however, the most critical aspect is how to take these results and use them to enhance or develop an efficient protocol in recalcitrant species that will generate sufficient embryos for breeding or basic research purposes.

### Tissue Culture-Based Protocol Expansion

Traditional and less-traditional tissue culture additives used to promote androgenesis have been reviewed previously (Niazian and Shariatpanahi, 2020). Thus, we focus on a few tissue culture additives that have proven effective in recalcitrant systems, which may be useful for protocol expansion to additional genotypes and species. Arabinogalactan proteins (AGPs), polyamines, and epigenetic modifiers will be reviewed briefly here. A recent book compiled of 62 chapters including detailed DH protocols for 44 species has been published and is a useful resource for DH methodology (Seguí-Simarro, 2021).

#### Arabinogalactan Proteins and Extracellular Matrices

Extracellular matrices secreted by cell cultures may contain clues for protocol expansion. Borderies et al. (2004) observed the formation of an extracellular matrix in the culture of maize microspores, consisting of secreted AGPs, cell wall invertase, thaumatin, β-1,3-glucanase, and chitinase. Rye anther cultures exhibited high activities of β-1,3-glucanases and chitinases in androgenic embryos, and AGPs were found to accumulate in the vesicles and inner walls of the anthers (Zieliński et al., 2021). *Brassica* microspores and pollen actively produce and secrete AGPs (El-Tantawy et al., 2013). Testillano (2019) reported that AGPs, cell wall remodeling, and pectin demethylesterification were essential features for androgenesis. Niazian and Shariatpanahi (2020) identified programmed cell death in a subpopulation of cultured microspores as leading to the secretion of the matrix, and the AGPs in the matrix coordinated with cell wall remodeling. In addition, the extracellular matrix contained polyamines and antioxidants to ameliorate stress.

Rodríguez-Sanz et al. (2014) found that cultures of androgenic embryos and zygotic embryos of *Quercus* shared several similarities, including cell wall remodeling by pectin esterification, DNA hypomethylation, and an increase in auxin. AGPs and pectin demethylesterification were deemed essential for somatic embryogenesis in *Quercus* (Pérez-Pérez et al., 2019). Leljak-Levanić et al. (2015) noted similarities between somatic embryos and zygotic embryos of several species, including the formation of an extracellular matrix containing polysaccharides and AGPs, and pathogenesis-related (PR) β-1,3-glucanases and chitinases. A fibrous- and vesicle-rich matrix was essential for somatic embryogenesis. AGPs were found to be secreted in somatic embryogenic but not in non-embryogenic cultures of hybrid *Abies* (Šamaj et al., 2008), and AGPs from one cereal genus stimulated zygotic embryo development of another cereal genus (Paire et al., 2003). AGPs released in cultures act as signaling molecules (Hernández-Sánchez et al., 2009). These findings taken together indicate that plant embryos – whether zygotic, somatic, or androgenic in origin – secrete AGPs that coordinate with cell wall modifying enzymes to direct cells into an embryogenic pathway and/or maintain embryogenesis.

Letarte et al. (2006) reported improvement of wheat isolated microspore culture response with the use of a commercial source (10 mg/L Larcoll) of arabinogalactans (AGs) and gum Arabic (10 mg/L) as a source of AGPs added to the culture medium. Similarly, addition of 10 mg/L gum Arabic promoted microspore embryogenesis in white cabbage (*Brassica oleracea* var. *capitata*, Yuan et al., 2012) and eggplant (*Solanum melongena*, Corral-Martinez and Segui-Simarro, 2014). Kale (*Brassica oleracea* var. *sabellica*) microspore embryogenesis was enhanced with the addition of 10 mg/L AGs (Niu et al., 2015), and 10 mg/L gum Arabic promoted androgenesis in barley anther cultures (Makowska et al., 2017). Ovary co-culture combined with the addition of AGPs further enhanced wheat microspore embryogenesis (Coskun and Savaskan, 2017). Pepper (*Capsicum*) microspore response improved with addition of 120 mg/L gum Arabic (Pourmohammad et al., 2021). Evidently, the amount, source, and quality of AGs and AGPs required by different species may differ, but addition of AGs/AGPs shows promise for extending androgenesis protocols to additional genotypes and species.

To our knowledge, application of chitinases or glucanases have not been reported in androgenesis protocols.

#### Polyamines

Cha-um et al. (2009) included 0.5 mm spermidine in the culture of rice anthers with an improvement in androgenic response. Redha and Suleman (2011) pretreated wheat anthers with 1 mm putrescine or spermine for 1 h prior to culture with an improved androgenic response. In contrast, *Brassica* isolated microspore cultures utilized 0.5–1.0 mg/L putrescine (micromolar range) with beneficial results, but higher concentrations were inhibitory (Ahmadi et al., 2014; Bhatia et al., 2021). Similarly, pepper isolated microspores were exposed to 0.5–1.0 mg/L putrescine during the temperature stress and sugar starvation stages of the protocol with improved androgenic response, and higher concentrations were inhibitory (Heidari-Zefreh et al., 2019). The much lower effective concentrations of polyamines in isolated microspore culture are likely due to their direct availability to androgenic cells, whereas anther cultures have multiple layers of cells to penetrate in order to access the androgenic cell (e.g., the microspore).

#### Epigenetic Additives

The understanding of the embryogenesis process and the identification of epigenetic reprogramming has led to incorporating additives (enzyme inhibitors) that are involved in epigenetic reprogramming. Histone deacetylase inhibitors (i.e., Trichostatin-A, scriptaid, sodium butyrate) have been shown to enhance embryogenesis in several species. Trichostatin-A has been shown to induce embryogenesis in *B. napus*, *B. rapa*, and wheat (Li et al., 2014; Zhang et al., 2016; Jiang et al., 2017; Wang et al., 2019; Castillo et al., 2020). Concentrations ranged from 0.008 to 0.4 μm depending on the species as well as the method of incorporation (short incubation period or kept continuously in culture). Not only was embryogenesis increased, but there was an increase in green plant production in wheat (Jiang et al., 2017; Wang et al., 2019). Scriptaid, another histone deacetylase inhibitor, has also resulted in an increase in embryogenesis and green plant production in wheat, however the plant morphology was classified as abnormal (Wang et al., 2019). In both spring wheat and winter wheat, the addition of sodium butyrate did not show any added benefit in inducing microspore embryogenesis, as embryo numbers decreased (although embryos appeared to be of better quality with a higher regeneration rate; Kathiria et al., 2016; Wang et al., 2019).

The histone methyltransferase inhibitor, BIX-01294, has also been evaluated in microspore culture of *B. napus* and barley (Berenguer et al., 2017). Embryo production was highest with the BIX-01294 treatment of 1.0 or 2.5 μm for 4–6 days, however detrimental effects were observed with long term treatment (30 days). The addition of 5-azacytidine (5-AzaC), inhibitor of DNA methyltransferase, to barley and *B. napus* microspores showed that a 4 day treatment of 2.5 μm AzaC increased embryo induction, but longer treatments were detrimental to embryo production (Solís et al., 2015). Other studies in triticale indicated that the addition of 5-AzaC or 2′-deoxy-5-azacytidine was not able to overcome the recalcitrant nature of the low responding line but was able to enhance embryo production and plant regeneration in the highly embryogenic line (Nowicka et al., 2019).

#### Advances in Embryo Conversion

Some species (e.g., *Brassica*) undergo microspore embryogenesis through to conversion into plantlets readily, while others (e.g., *Capsicum*) exhibit induction of microspore embryogenesis but significant bottlenecks at the conversion stage (Ferrie and Caswell, 2011). Recent advances in the conversion of pepper microspore embryos may provide a strategy for extending conversion success to other species.

One of the best protocols for conversion of pepper plants from anther cultures was described by Dolcet-Sanjuan et al. (1997). A two-layer culture system was used with a semisolid medium underlayer and a liquid medium overlay based on NN (Nitsch and Nitsch, 1969) using 2% maltose as sugar source, except the underlayer medium included 0.5% activated charcoal (AC) as well as gelling agent. Cultures were incubated in dark at 7°C for 1 week then moved to 28°C for 8 week Cultures were transferred to fresh semisolid medium with 2% maltose but lacking AC for 3–4 week in light conditions, then finally to fresh medium with 2% sucrose for plantlet recovery.

This anther-based bilayer protocol was adapted to the shed-microspore culture of pepper by Supena et al. (2006a,b) and Supena and Custers (2011). The shed-microspore cultures were incubated in the dark at 9°C for 1 week, then 28°C for 3 week, then 21°C for 4 week prior to exposure to light. The underlayer semisolid medium was based on NN with 2% maltose and 1.0% AC. The overlayer liquid medium lacked AC. After 4 week incubation (when temperature was reduced), the liquid overlayer medium was supplemented with 2.5 μm zeatin and 5.0 μm indole acetic acid. These modifications resulted in enhanced pepper microspore embryo yield and quality.

### Progress in Androgenesis of Recalcitrant Grain Legumes

The legume species are considered recalcitrant in tissue culture. Pratap et al. (2018) stated that slow morphogenesis, albinism, genotypic specificity, and vitreous tissues can hinder plant regeneration from cells and tissues. Croser et al. (2006) reviewed DH progress within the Fabaceae and stated that DH technology is not being used in breeding programs for any leguminous species because of the recalcitrant nature of the family. There have been a few reports of anther culture and isolated microspore culture with legumes but there are no efficient, routine methods. Bayliss et al. (2004) cultured isolated microspores of several species of lupin (*Lupinus* spp.) and reported the production of pro-embryos, but there was no further development. There have been some positive initial results on isolated microspore culture of field pea (*Pisum sativum* L.; Ochatt et al., 2009; Bobkov, 2018). Microcalli (Bobkov, 2018) and embryogenic/organogenic calli (Ochatt et al., 2009) resulted in a few (8) embryos; however, these were of poor quality and did not survive transfer to soil (Ochatt et al., 2009).

Legumes are recalcitrant to androgenesis, especially the commercially important grain legumes. Recent progress in chickpea and soybean bode well for resolving this challenge. Panchangam et al. (2014) induced chickpea anthers with cold stress and cultured them on a high osmoticum medium containing high auxin. After 4 day of culture, microspores were observed with 3–5 nuclei from all four genotypes tested, and these were subjected to expressed sequence tag (EST) analysis. Two members of the Agamous-like (AGL) transcription factor family, AGL-16 and AGL-6, were among the chickpea ESTs, which coincide with androgenesis activity in *Arabidopsis*. Abdollahi and Rashidi (2018) cultured chickpea anthers on medium containing 10 mg/L 2,4-D and 15–25 mg/L AgNO_3_ and successfully recovered haploid plants. Grewal et al. (2009) combined several stress treatments, including cold shock (4°C for 4 day), electric pulse (125–200 V), centrifugation (168 *g* for 10 min), and high osmotic stress (563 mmol) to generate embryogenic structures. From these experiments (Grewal et al., 2009; Abdollahi and Rashidi, 2018), 52 plants survived and were transferred to soil. Many of the chickpea androgenesis experiments have focused on anther culture methods; however isolated microspore culture is often the preferred method, as it can be more efficient than anther culture. Isolated microspore culture of chickpea have resulted in early stage embryo development (Croser et al., 2011), but there was no further development. If this protocol can be enhanced, doubled haploid breeding will be feasible for chickpea.

Similarly, a two-step donor plant cold temperature stress defined using soybean anther cultures (Garda et al., 2020) elicited characteristic responses from isolated microspores at the cellular (Hale et al., 2021) and molecular (Hale et al., 2020) levels, similar to model systems. Nitrogen starvation, but not sugar starvation (imposed using maltose instead of sucrose), enhanced androgenic response in anther cultures (Garda et al., 2020). Phytohormones were required for isolated microspores to undergo sustained cell divisions, but anther cultures did not require phytohormone supplementation (Garda et al., 2020). Soybean isolated microspore cultures exhibited matrix formation, and the matrix contained AGPs (Hale et al., 2020, 2021). While conversion into plants has yet to be achieved, the soybean induction protocol presents a significant advancement for this crop.

## Conclusion

In 1964 the first report was published on the regeneration of plants from anther culture (Guha and Maheshwari, 1964). Now, planst breeders are routinely using these methods to develop new cultivars. For example, the majority of the canola breeding organizations in Canada are utilizing the DH methods and most of the varieties are DH. As outlined, these DH methods have been instrumental in speeding up the breeding program in many crops. We have also been able to acquire some understanding of the molecular and biochemical processes that are involved in the induction of the microspore to change the developmental process to one of embryo development. These haploid cells can be manipulated through mutagenesis, transformation, or gene editing to generate variation and generate lines with the desired trait of interest.

Despite these successes, there are still many commercially important crops that are considered recalcitrant when it comes to DH methodology. There may be a few publications indicating microspore induction, early stages of embryo development, or even plant development, but a routine protocol that would yield sufficient embryos and subsequent plants for breeding purposes is not available.

## Author Contributions

All authors contributed equally to this work. BH and GP wrote the abstract. BH wrote the introduction, incorporated edits, modified writing style, and constructed reference section. SC wrote Section “Plant Breeding Applications: From Lab to Field.” JS wrote Section “Applications for Gametophyte Reprogramming.” GP and AF wrote Section “Expansion of Protocols Across Genotypes and Taxa.” AF wrote Section “Conclusion.” BH, AF, and SC edited the manuscript.

## Funding

This study was funded through support from USDA-NIFA Non-Land Grant Colleges of Agriculture Capacity Building award number 2018-70001-28762 and Corteva Agriscience Open Innovation.

## Conflict of Interest

The authors declare that this review was written in the absence of any commercial or financial relationships that could be construed as a potential conflict of interest.

## Publisher’s Note

All claims expressed in this article are solely those of the authors and do not necessarily represent those of their affiliated organizations, or those of the publisher, the editors and the reviewers. Any product that may be evaluated in this article, or claim that may be made by its manufacturer, is not guaranteed or endorsed by the publisher.
